# Antiviral Drug- and Multidrug Resistance in Cytomegalovirus Infected SCT Patients

**DOI:** 10.1016/j.csbj.2015.01.003

**Published:** 2015-02-10

**Authors:** Katharina Göhring, Klaus Hamprecht, Gerhard Jahn

**Affiliations:** Institute of Medical Virology and Epidemiology of Viral Diseases, University Hospital of Tübingen, 72076 Tübingen, Germany

**Keywords:** HCMV, Multidrug resistance, Antivirals, Stem cell transplantation, Cytomegalovirus

## Abstract

In pediatric and adult patients after stem cell transplantation (SCT) disseminated infections caused by human cytomegalovirus (HCMV) can cause life threatening diseases. For treatment, the three antivirals ganciclovir (GCV), foscarnet (PFA) and cidofovir (CDV) are approved and most frequently used. Resistance to all of these antiviral drugs may induce a severe problem in this patient cohort. Responsible for resistance phenomena are mutations in the HCMV phosphotransferase-gene (UL97) and the polymerase-gene (UL54). Most frequently mutations in the UL97-gene are associated with resistance to GCV. Resistance against all three drugs is associated to mutations in the UL54-gene. Monitoring of drug resistance by genotyping is mostly done by PCR-based Sanger sequencing. For phenotyping with cell culture the isolation of HCMV is a prerequisite. The development of multidrug resistance with mutation in both genes is rare, but it is often associated with a fatal outcome. The manifestation of multidrug resistance is mostly associated with combined UL97/UL54-mutations. Normally, mutations in the UL97 gene occur initially followed by UL54 mutation after therapy switch. The appearance of UL54-mutation alone without any detection of UL97-mutation is rare. Interestingly, in a number of patients the UL97 mutation could be detected in specific compartments exclusively and not in blood.

## 1 Introduction

The Human Cytomegalovirus (HCMV) belongs to the Betaherpesvirus family and leads, after primary infection, to a lifelong latency. Under special condition such as immunosuppression, the virus can be reactivated and may lead to severe diseases. The HCMV genome comprised about 230 kb with more than 250 open reading frames. Transplant recipients have a high risk to develop a primary infection or recurrent infection with human cytomegalovirus (HCMV) [1]. The incidence depends on different risk factors as serostatus, duration of treatment and immunosuppressive regimen. Antiviral drugs for the therapy of disseminated HCMV infection are ganciclovir (GCV), cidofovir (CDV) and foscarnet (PFA) [2]. All three drugs target the UL54-gene which encodes for the viral DNA polymerase. GCV has to be phosphorylated by the viral phosphotransferase UL97. Mutations in both genes can lead to an antiviral resistance. Because GCV is the drug of choice for primal therapy, most resistance mutations can be detected in the UL97 gene. In some cases additional mutation in the UL54 gene can be detected after a therapy switch to either PFA or CDV.

The development of multidrug resistant HCMV infection and disease with mutations in the UL97- and the UL54-gene after allogeneic hematopoietic cell transplantation (HCT) can be life-threatening. Therefore a rapid and close meshed monitoring of HCMV infection and viral load under therapy after HCT is mandatory [3–5].

Diagnosis of resistant HCMV infection is mostly performed by genotypic assays of UL97 and UL54 genes. A basic problem is the interpretation of unknown or rarely reported point mutations. To concern this, Marker Transfer Analysis is needed and a web-based data base for HCMV drug resistance mutations was established [6].

New compounds with different mechanism of action are under investigation [7–9]. The clinical effectiveness of Maribavir is still investigated in clinical trials and is not commercially available up to now [10–12]. This short review will focus on the development of drug- and multidrug resistance and the characterization of newly detected mutations.

## 2 The UL97 Open Reading Frame

**Fig. 1:**
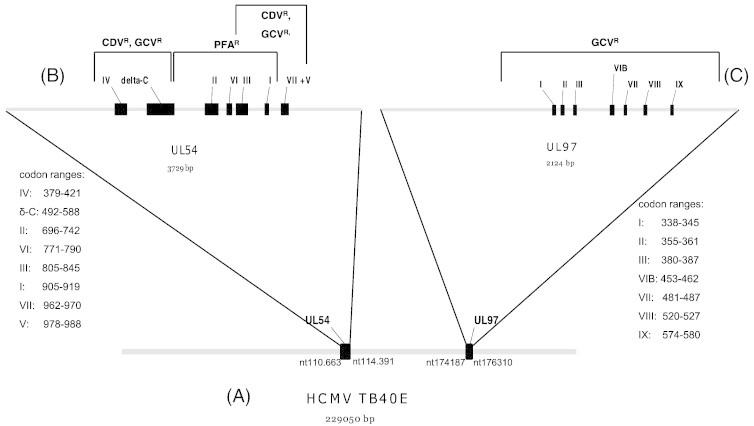
(A) Position of the UL54 and the UL97 open reading frame in the genome of HCMV strain TB40E [50]. (B) UL54 open reading frame with conserved subdomains (black boxes) of the gene. Mutations can lead to resistance to GCV, CDV or PFA and can also cause multidrug resistance. (C) UL97 open reading frame including the conserved subdomains (black boxes). Mutations in the UL97 gene can cause resistance to GCV.

The UL97 gene encodes for the viral phosphotransferase. Beside many other targets like for example pUL44, pUL69 [13] or IFI16 [14], it is responsible for the phosphorylation of GCV. The UL97-protein contains several conserved subdomains with specific functions (Fig. 1). The subdomain I is responsible for ATP binding, subdomains II, III, VIB and VII are involved in the phosphate transfer and subdomain IX is essential for substrate binding. Mutations in the UL97 gene might cause resistance to GCV [13].

## 3 The UL54 Open Reading Frame

The UL54 gene of HCMV encodes for the viral DNA polymerase. Like a wide range of DNA polymerases, the UL54 protein has conserved functional regions (Fig. 1). Mutations in the HCMV polymerase gene might cause resistance to GCV, CDV and PFA and can also generate multidrug resistance phenotypes [13].

## 4 Risk Groups and Factors

Risk groups for the development of disseminated HCMV diseases are patients with impaired immune system including patients with AIDS, transplant recipients, congenitally HCMV-infected premature infants and patients with immune deficiency, but prevalence data are limited. The risk to develop a resistant HCMV infection in transplant patients depends on different factors such as serostatus, type of transplant and the level of immunosuppression [7]. For the prevention of HCMV disease in transplant patient, two different strategies are used. Prophylactic treatment using valganciclovir seems to be a common strategy in organ transplant recipients [1]. Patients after stem cell transplantation have a high risk to develop a severe HCMV disease [13]. This is due to a high immunosuppressive therapy and to long term donation of antiviral drugs. Normally these patients received pre-emptive therapy [15]. The development of a resistant HCMV infection can be observed after prolonged drug exposure over weeks or months with the greatest risk for CMV seronegative patients after solid organ transplantation receiving an organ from a seropositive donor with a frequency of 5–10% [16]. In kidney transplant recipients the incidence increased up to 12.5% and seems to be still higher in lung transplant recipients [13,17–19]. Besides antiviral potency, bioavailability and effectiveness of drug delivery are important factors for the development of antiviral drug resistance [13]. This is also of importance in the context of the development of antiviral drug resistance in different compartments.

## 5 Antiviral Therapy

Therapy against HCMV diseases is due to three antiviral drugs. The nucleoside analogue ganciclovir (GCV) is used as gold-standard. The L-valyl ester of ganciclovir valganciclovir (ValGCV) can be administrated orally and is rapidly absorbed and hydrolyzed to ganciclovir [7]. It is phosphorylated by the viral UL97-gene product and cellular kinases and is afterwards incorporated in the growing DNA-chain and leads to elongation stop. Adverse effects of ValGCV and GCV are neutropenia and teratogenic and hematotoxic effects [18]. Cidofovir (CDV), a nucleotide analogue, is phosphorylated by cellular kinases and stops also chain elongation, since viral DNA polymerase is the final target molecule. It has severe secondary effects like nephrotoxicity and neutropenia which limits the clinical application. The pyrophosphate analogue foscarnet (PFA) interacts directly with the viral polymerase for the pyrophosphate binding site, encoded by the UL54 open reading frame, and inhibits the enzyme reversible [20–22]. The treatment with PFA showed many adverse effects like electrolyte abnormalities, anemia and granulocytopenia that limit the clinical use of the drug [18]. Mutations in the HCMV phosphotransferase-gene (UL97) and the polymerase gene (UL54) are responsible for resistance to ganciclovir (GCV), cidofovir (CDV) and foscarnet (PFA). Over 90% of all GCV-resistant clinical isolates do have mutations in the UL97 gene [2,7]. Special mutations in conserved subdomains of the UL54-gene can cause resistance to GCV, CDV and PFA as well as cross-resistance between all three antiviral drugs. Resistance to Maribavir is mediated by mutations in the UL97- and the UL27 gene [8,13,22].

## 6 Laboratory Diagnostics

### 6.1 Genotypic Assays

The gold standard and mostly used genotypic method for the detection of resistant HCMV strains is Sanger sequencing of PCR products from the UL97 and the UL54 genes [13]. Different new techniques have been established [23]. Deep sequencing methods allow the detection of minorities of mutations in a patient isolate mixture [24,25]. However, the importance of minor subpopulations in context of treatment remains unclear [26].

Other methods for the detection of drug resistance associated mutations are restriction fragment length polymorphism (RFLP) and real time PCR assays. Both methods allow a detection of resistant mutations in the UL97 gene. The RFLP is based on restriction endonuclease recognition sites in PCR products of the UL97 gene. Mutations result in gain or loss of these restriction sites and in comparison to a wildtype control, detection of mutations can be performed by digest of the PCR product followed by agarose gel electrophoresis. With different enzymes a pattern of fragments could be compared. Other advantages are that amplification can be made directly from material of the patient; it is fast, cost saving, easy to perform and mixtures of wildtype and mutant strains can be detected. Nevertheless, the RFLP analysis is restricted to known mutations that lead to a gain or loss of restriction sites. New mutations cannot be detected using this method [27–32], and an appropriate assay for detection of gain or loss of a defined point mutation has to be available. The most important canonical UL97 mutations are detectable via RFLP (recognition site), like M460V (NlaIII), M460I (Mse I) [47], A591V (HaeIII), C592G (FseI) [90,91], A591V/C592G (HaeIII/Fse I/Taq I) [47], A594V (Hin6I), L595S (Taq I), L595F (Mse I) [29], C603W [91], and C607Y [71]. RFLP assays for HCMV UL54 have no diagnostic value, since they are distributed over the whole gene.

Real-time PCR using melting curve analysis for the detection of specific UL97-mutation is a very fast and sensitive method. It also allows a semiquantitative detection of wildtype and mutant mixtures out of patient material and a simultaneously detection of different mutations in the same approach. Equally to RFLP analysis, the real time PCR approaches can only detect known mutations. The assays cannot distinguish between different mutations and polymorphisms near known mutations can influence the melting curve [33,34]. Nevertheless, these assays are a good tool for a rapid screening of the most common UL97-mutations out of patient materials.

### 6.2 Phenotypic Assays

The phenotypic characterization of isolates extracted from patient materials is very time consuming for HCMV and has only restricted use in routine diagnostics, since it is prerequisite to get a viral isolate. Nevertheless if a viral isolate is available, it is possible to check beside GCV the susceptibility also to PFA and cidofovir. However it remains important in cases with unclear genotypic results or detecting of new mutations.

Different types of Plaque reduction assays (PRAs) were described which are based on the determination of a required drug concentration necessary to reduce viral growth in cell culture, the ID_50_ (inhibitory dose 50%) values which define the halfmaximal reduction of the number of HCMV plaques resulting from in vitro culture without anitiviral compound. Standardization of these PRAs is still a problem, especially in context of definitions of cut-off levels for resistance, which also depends on the availability of suitable therapy-naïve wild-type virus strains. Nevertheless, these assays are necessary to confirm new mutations and to clarify unclear genotypic results [35–38].

An interesting tool for the correlation of genotypic results to a specific potentially resistant phenotype was published by Chevillotte et al. [39]. Comparable to databases for HBV or HIV drug resistance, the platform allows a correlation of Marker Transfer verified UL97- and UL54 mutations to a correspondent phenotype.

## 7 Marker Transfer

Marker Transfer Analysis comprised the incorporation of a not described UL97- or UL54- mutation in the wildtype background of HCMV followed by o phenotypic characterization of the resulting recombinant HCMV strain in comparison to the wildtype. The large and complex genome of HCMV was a huge problem in early time period of antiviral testing. Using homologous recombination by cotransfection, multiple segments of HCMV-DNA was cotransfected into HFF [29,40]. One segment contained the mutation which should be characterized; the other segments were wildtype HCMV DNA. The resulting virus had to be propagated and finally plaque purified. Recombination events are normally very rare and cannot be controlled in cell culture. Therefore the generation of HCMV mutant using this method was time consuming and often not successful. Also the co-transfection of cosmid clones was very hard to perform [41].

A milestone for the generation of recombinant HCMV mutants was the development of HCMV as bacterial artificial chromosome (BAC). Different methods for the reconstitution of HCMV-BAC mutants were described [42–44]. BAC clones are very stable and the mutagenesis can be performed in specific *Escherichia coli* strains where the recombination of HCMV BAC with a PCR product containing the unknown mutation, can be controlled. The most effective one was published by Tischer et al. [45,46] and allows the generation of HCMV mutants in *E.coli* without any foreign sequences. The resulting BAC mutant DNA is then transfected into HFF and after propagation, phenotypic characterization can be performed. The characterization of the recombinant HCMV-BACs is normally done using PRA.

## 8 Multidrug Resistance

Multidrug resistance in patients under antiviral treatment of HCMV diseases is described by different groups [3,4,39,48,49]. The appearance of resistant phenotype against all available drugs often leads to a fatal outcome.

Typically, multidrug resistance phenotypes appear after long term treatment span weeks or months. Normally therapy of a HCMV disease with a positive DNAemia in whole blood or plasma starts with the donation of GCV as drug of choice. In most cases the viral load decreases. Mutations in the UL97 gene can be observed shortly before a first viral peak value. After a therapy switch to either PFA or CDV the viral load decreases again. After weeks of treatment, additional UL54 mutations can be observed before the viral load increases again [48]. Isolated UL54-mutations leading to a resistance phenotype are rarely reported. Phenotypic assay normally confirms and completes the genotypic results. Newly detected mutations have to be confirmed by Marker Transfer Analysis. Also combination of different mutation In UL97- and UL54 gene have to be investigated [48]. The mutations characterized up to now are illustrated in Tables 1 and 2.

## 9 Summary

The development of drug- or multidrug resistant HCMV infection in patients after SCT might cause severe clinical problems. Mutations in the UL97 can lead to resistance to GCV while mutations in the UL54 gene region can cause resistance to all three antiviral drugs GCV, CDV and PFA. Since GCV is the first choice for treatment, mutations in the UL97 gene appear first followed by UL54 mutations after therapy switch resulting in a multidrug resistant phenotype.

There are limited reports about the development of multidrug resistance in transplant recipients [3,4,39,48,49]. Multidrug resistant HCMV infection can cause potential lethal HCMV disease. Close monitoring of HCMV reactivation by PCR and to follow up the viral load under therapy after HCT as well as after organ transplantation is mandatory. The role of specific combinations of UL97- and UL54-mutations as well as polymorphisms associated mutations have to be further analyzed with regard to the clinical outcome and treatment failure.

## Figures and Tables

**Table 1:** Resistance mutations in the UL97 gene confirmed by Marker Transfer or recombinant phenotype.

Mutation	GCV ratio	References
L405P	2.5	[46]
M460I	5	[31,47–52]
M460T	9.3	[46]
M460V	8.3	[22,25,31,49–56]
V466G	3.5	[57]
H520Q	10	[22,25,31,49,54,58,59]
Del591–594	3–10	[22,25,34,49]
Del591–607	6.2	[49]
C592G	2.9	[50,51,53,55]
A594E	3.0	[46]
A594G	13.5	[50,61]
A594P	na	[31,48,50,60]
A594T	2.7	[22,25,31,48–51]
A594V	8.3	[22,25,31,48,49,51–54,59]
L595F	15.7	[49–52]
L595S	9.2	[22,25,31,48,49,51,53–55,85]
L595W	5.1	[31,49,54]
L595del	13.3	[62]
Del595–603	8.4	[63]
E596G	2.3	[48,49]
E596Y	6.4	[69]
G598S	na	[64]
K599T	5.3	[65]
L600del	1.9	[49]
T601del	Nq	[66]
Del601–603	11	[56]
C603R	3.6–8.3	[31,46,57]
C603S	1.9	[31,46]
C603W	8	[22,31,46,48–51,59,85]
C607F	1.9	[22,49,50]
C607Y	12.5	[31,49,50,67]
I610T	2.6	[69]
A613V	2.3	[68]
L405P	2.5	[51]
M460I	5	[37,52–54,18,55,56]
M460T	9.3	[51]
M460V	8.3	[18,28,31,37,54–60]
V466G	3.5	[61]
C518Y	12	[92]
H520Q	10	[28,31,37,54,58,61,46]
Del591–594	3–10	[28,31,40,54]
Del591–607	6.2	[54]
C592G	2.9	[18,55,57,59]
A594E	3.0	[51]
A594G	13.5	[18,65]
A594P	Na	[18,37,53,64]
A594T	2.7	[18,28,31,37,53–55]
A594V	8.3	[28,31,37,46,53–58]
L595F	15.7	[18,54–56]
L595S	9.2	[28,31,37,53–55,57–59,89]
L595W	5.1	[37,54,58]
L595del	13.3	[66]
Del595–603	8.4	[67]
E596G	2.3	[53,54]
E596Y	6.4	[73]
G598S	Na	[68]
K599T	5.3	[69]
L600del	1.9	[54]
T601del	Nq	[70]
Del601–603	11	[60]
C603R	3.6–8.3	[37,51,61]
C603S	1.9	[37,51]
C603W	8	[18,28,37,46,51,53–55,89]
C607F	1.9	[18,28,54]
C607Y	12.5	[18,37,54,71]
I610T	2.6	[73]
A613V	2.3	[72]

**Table 2:** Resistance mutations in the UL54 gene confirmed by Marker Transfer. Resistance ratios are marked in bold numbers.

Mutation	GCV ratio	PFA ratio	CDV ratio	*References*
D301N	**2.6**	0.5	**3**	[70]
N408D	**4.9**	1.3	**5.6**	[35]
N408K	**4.2**	0.7	**21**	[71]
N410K	**2.9**	0.8	**3**	[70]
F412C	**4.2**	1.2	**18**	[72]
F412V	**4.3**	1.1	**15.5**	[35]
D413A	**6.5**	0.8	**11**	[56]
D413E	**4.8**	0.8	**4.3**	[70,87]
N495K	1.1	**3.4**	1.1	[73]
L501I	**6**	1.4	**9.1**	[35]
T503I	**2.9**	0.5	**6.1**	[70]
K513E	**5**	1.4	**9.1**	[35]
K513N	**6**	1.1	**12.5**	[74]
D515E	**2.4**	1.1	1.6	[69]
L516R	**2.1**	0.8	**5.1**	[70]
I521T	**3.1**	0.9	**3.9**	[75]
P522A	**3**	1	**4.1**	[75]
P522S	**3.1**	1.1	**3.6**	[35]
L545S	**3.5**	1.2	**9.1**	[35]
D588E	1.3	**2.3**	1.1	[35,77]
D588N	**3.8**	**3.2–9**	**2.7**	[76]
T700A	0.9	**4.7**	1.5	[78]
V715M	1	**5.5**	1.1	[78]
E756D	1.2	**3.4**	0.7	[70]
E756K	**3.5**	**> 8**	**2.2**	[70,87]
E756Q	1.7	**4.3**	1	[79]
L776M	**2.5**	**3.5**	1	[80]
V781I	**1–4**	**4–5.2**	1.2	[35,76]
V787L	**2.4**	**4.1**	1	[79]
L802M	**1.1–3.5**	**3.2–10.8**	0.9–1.8	[35,72]
K805Q	1	0.18	**2.2**	[35]
A809V	**2.6**	**6.3**	1.7	[81]
V812L	**2.5**	**2.9**	**3.2**	[74]
T813S	**2.5**	**4.9**	**2.7**	[82]
T821I	**4.5**	**21**	1.9	[35]
A834P	**5.4**	**6.4**	**3**	[71]
T838A	1.8	**2.4**	0.8	[77]
G841A	**3.2**	**4.3**	**2.6**	[82]
Del918–982	**8.3**	**3.6**	**2.8**	[83]
A987G	**5.3**	1.2	**11.3**	[84]
D301N	**2.6**	0.5	**3**	[74]
N408D	**4.9**	1.3	**5.6**	[41]
N408K	**4.2**	0.7	**21**	[75]
N408S	**3.1**	Nd	**7.5**	[92]
N410K	**2.9**	0.8	**3**	[74]
F412C	**4.2**	1.2	**18**	[76]
F412V	**4.3**	1.1	**15.5**	[41]
F412L	**4.6**	1.1 ×	**9.4× **	[93]
F412S	**5.3**	0.8 ×	**13× **	[93]
D413A	**6.5**	0.8	**11**	[77]
D413E	**4.8**	0.8	**4.3**	[74,91]
P488R	**3.5**	Nd	**7.9**	[93]
N495K	1.1	**3.4**	1.1	[77]
L501I	**6**	1.4	**9.1**	[41]
T503I	**2.9**	0.5	**6.1**	[74]
K513E	**5**	1.4	**9.1**	[41]
K513N	**6**	1.1	**12.5**	[78]
D515E	**2.4**	1.1	1.6	[73]
L516R	**2.1**	0.8	**5.1**	[74]
I521T	**3.1**	0.9	**3.9**	[79]
P522A	**3**	1	**4.1**	[79]
P522S	**3.1**	1.1	**3.6**	[41]
C539R	**3.2**	Nd	**13.3**	[93]
L545S	**3.5**	1.2	**9.1**	[41]
L545W	**5.9**	1.3 ×	**6.3× **	[93]
Q578H	**3.3**	**4.5**	**2.3**	[93]
Q578L	1.9	**3.0**	0.8	[93]
D588E	1.3	**2.3**	1.1	[41,81]
D588N	**3.8**	**3.2–9**	**2.7**	[80]
T700A	0.9	**4.7**	1.5	[82]
V715M	1	**5.5**	1.1	[82]
E756D	1.2	**3.4**	0.7	[74]
E756K	**3.5**	**> 8**	**2.2**	[74,91]
E756Q	1.7	**4.3**	1	[83]
L776M	**2.5**	**3.5**	1	[84]
V781I	**1–4**	**4–5.2**	1.2	[41,80]
V787L	**2.4**	**4.1**	1	[83]
L802M	**1.1–3.5**	**3.2–10.8**	0.9–1.8	[41,76]
K805Q	1	0.18	**2.2**	[41]
A809V	**2.6**	**6.3**	1.7	[85]
V812L	**2.5**	**2.9**	**3.2**	[78]
T813S	**2.5**	**4.9**	**2.7**	[86]
T821I	**4.5**	**21**	1.9	[41]
A834P	**5.4**	**6.4**	**3**	[75]
T838A	1.8	**2.4**	0.8	[81]
G841A	**3.2**	**4.3**	**2.6**	[86]
Del981–982	**8.3**	**3.6**	**2.8**	[87]
A987G	**5.3**	1.2	**11.3**	[88]
